# Glaucoma and mortality risk: findings from a prospective population-based study

**DOI:** 10.1038/s41598-021-91194-3

**Published:** 2021-06-03

**Authors:** Tilman Kühn, Sabine Rohrmann, Nena Karavasiloglou, David S. Friedman, Aedin Cassidy, Till Bärnighausen, Alexander K. Schuster, Stefan Nickels

**Affiliations:** 1 Institute for Global Food Security, Queen’s University Belfast, Belfast, UK; 2 Heidelberg Institute of Global Health, University of Heidelberg, Heidelberg, Germany; 3 Division of Chronic Disease Epidemiology, Epidemiology, Biostatistics and Prevention Institute (EBPI), University of Zurich, 8091 Zurich, Switzerland; 4 Massachusetts Eye and Ear, Harvard Medical School, Boston, MA USA; 5 Department of Ophthalmology, University Medical Center Mainz, Mainz, Germany; 6 Darmstadt, Germany

**Keywords:** Eye diseases, Diseases, Risk factors

## Abstract

Glaucoma is a neurodegenerative disease with a structural change of the optic nerve head, leading to visual field defects and ultimately blindness. It has been proposed that glaucoma is associated with increased mortality, but previous studies had methodological limitations (selective study samples, lack of data on potential confounders, self-reported or secondary data on glaucoma diagnoses). We evaluated the association between diagnosed glaucoma and mortality in the population-based National Health and Nutrition Examination Survey (NHANES), a representative health survey in the United States. The survey cycles 2005–2006 and 2007–2008 included an extensive ophthalmic examination with fundus photography, which were used to derive standardized glaucoma diagnoses. Risk of all-cause mortality was assessed with multivariable Cox proportional hazards regression models accounting for the complex survey design of NHANES. Time to death was calculated from the examination date to date of death or December 31, 2015 whichever came first. 5385 participants (52.5% women) were eligible, of which 138 had glaucoma at baseline, and 833 died during follow-up. Participants with glaucoma were more likely to be older than those without glaucoma (mean age 69.9 vs. 56.0 years). Mean follow-up time was 8.4 years for participants with glaucoma, and 8.6 years for participants without glaucoma. Glaucoma was associated with increased mortality in an unadjusted Cox regression model (hazard ratio 2.06, 95% confidence interval 1.16 to 3.66), but the association was no longer statistically significant after adjusting for age and sex (hazard ratio 0.74, 95% confidence interval 0.46 to 1.17). Additional adjustment for a range of potential confounders did not significantly change the results. In this representative population-based study, we found no evidence of increased mortality risk in glaucoma patients.

## Introduction

Glaucoma is a chronic, progressive eye disease characterized by structural changes of the optic nerve head and visual field loss^1^. The number of people with glaucoma worldwide was estimated to be 64 million in 2013 and is expected to increase to 112 million by 2040^2^. Glaucoma is the most frequent cause of blindness worldwide^3,4^. The prevalence of glaucoma increases with age and varies depending on ethnic background: Black populations have the highest prevalence for open-angle glaucoma (POAG), the by far most common type of glaucoma, while Asian populations have the lowest^5,6^. Other factors associated with an increased risk for open-angle glaucoma are elevated intra-ocular pressure, male sex, high myopia and family history of glaucoma^1^. In the US, the prevalence of glaucoma in the age group 40 to 80 years is estimated to be 2.1%^7^.

Increased mortality rates have been observed among glaucoma patients in single cohort studies since the 1950s, although the mechanisms are unclear^8^. In 2009, a meta-analysis of nine studies showed no convincing evidence for an increased risk of all-cause mortality (Relative Risk [RR]: 1.13; 95% confidence interval [CI] 0.97–1.31) among those with POAG^9^. While there was a borderline significantly increased risk of cardiovascular mortality (RR: 1.20; 95% CI 1.00–1.43), this association was attenuated upon exclusion of a study based on self- or proxy-reported glaucoma (RR; 1.12; 95% CI 0.87–1.46)^9^. Inconsistent findings were reported from studies published after this meta-analysis. In a Scandinavian cohort with a follow-up duration of up to 30 years there was no evidence for an association of POAG with survival (hazard ratio [HR]: 1.04; CI 0.91–1.20)^10^. By contrast, a study from Taiwan analysing health insurance data from 30,000 adults showed that the risk of all-cause mortality was higher in patients with a new-coding of POAG compared to a control group without any ocular diseases (adjusted HR: 2.11; CI 1.76–2.54)^11^. In this study, over 80% of participants were younger than 65 years when glaucoma was first diagnosed, while glaucoma incidence increases with aging in population-based studies. In addition, the higher risk could be at least partly explained by the choice of a healthy comparison group, whereas other studies compared to participants without glaucoma. In an Indian rural cohort, POAG was associated with increased 10-year-mortality only in univariate analysis, but not in the adjusted model^12^.

Given the projected increase in glaucoma rates worldwide and the inconclusive evidence on mortality risks outlined above, well-conducted studies on life expectancy among glaucoma patients are still needed; if risks of overall and cardiovascular mortality were higher among glaucoma patients, the identification of underlying mechanisms and co-morbidities would be crucial for the delivery of targeted treatments^9,13^. Thus, we evaluated the association between glaucoma and mortality in the population-based National Health and Nutrition Examination Survey (NHANES). Unlike previous studies, NHANES provides representative, high-quality data on vision and eye health of the US adult population, with standardized and objective glaucoma assessments from the 2005–2006 and 2007–2008 survey cycles, as well as detailed information on health status, lifestyle and socio-economic background. Our goal was to analyze whether objectively and uniformly defined glaucoma, based on data that had initially been published in 2016^14^, was associated with mortality risk in a prospective set-up, when taking co-morbidities, such as diabetes or cardiovascular diseases, and other potential confounders into account.

## Results

### Sample description

Survey-weighted baseline characteristics of the study population are shown in Table 1. Out of 5385 eligible study participants 138 (1.7%) were diagnosed with glaucoma. These participants were older compared to those without glaucoma, with survey-weighted mean ages (standard errors) of 69.9 ± 1.9 vs. 56.0 ± 0.4 years at baseline. Overall, glaucoma patients showed a slightly less favourable pattern of socio-economic, lifestyle and health indicators. For example, they were less likely to have obtained at least a college degree (43.6% vs. 55.8%), less often reported to be never smokers (36.7% vs. 48.6%), but had a higher prevalence of diabetes (29.0% vs. 10.4%) compared to participants without glaucoma. With respect to race / ethnicities, the proportion of black people was higher among the participants with a glaucoma diagnosis (15.2% vs. 9.4%), while the proportion of white people was lower (73.7% vs. 77.3%). Importantly, it should be noted that differences with respect to education levels, marital status, health insurance membership, blood pressure, as well as smoking strongly depended on the age difference between the groups and became statistically non-significant upon adjustment for age. By contrast, the difference in the proportion of diabetes cases remained significantly higher among individuals with glaucoma upon adjustment for age (p = 0.004). BMI values were also significantly associated with glaucoma upon adjustment for age (p = 0.007).

**Table 1:** Survey-weighted general characteristics of NHANES 2005–2008 study participants with and without diagnosed glaucoma.

	Participants		*P* for difference^a^	
	With glaucoma (n = 138)	Without glaucoma (n = 5247)	Unadjusted	Age-adjusted
Age (years)	69.9 ± 1.9	56.0 ± 0.4	< 0.0001	–
Age group (%)				
Younger than 65 years	27.8	76.1		
65 years and older	72.2	23.9		
Sex (%)			0.64	0.30
Female	49.8	52.6		
Male	50.2	47.4		
Educational attainment (%)			0.04	0.56
College or higher	43.8	55.8		
High school or lower	56.2	44.2		
Ratio of family income to poverty, mean ± SE	3.1 ± 0.2	3.3 ± 0.1	0.29	0.52
Marital status (%)			0.03	0.29
Married/living with partner	56.1	69.9		
Not married/living with partner	43.9	30.1		
Race/ethnicity (%)			0.10	0.06
Mexican American	4.3	5.5		
Non-Hispanic Black	15.2	9.4		
Non-Hispanic White	73.7	77.3		
Other	6.8	7.8		
Health insurance, age < 65 years (%)			< 0.0001^b^	0.58^b^
None	10.4	16.7		
Private	65.7	65.0		
Government	0	6.8		
Private and government	23.9	11.5		
Health insurance, age ≥ 65 years (%)			< 0.0001^b^	0.58^b^
None	2.7	1.3		
Private	7.3	7.6		
Government	69.5	53.2		
Private and government	20.5	38.0		
Body mass index (kg/cm^2^)	29.7 ± 0.7	29.1 ± 0.2	0.34	0.007
Systolic blood pressure (mm/Hg)	131.2 ± 2.6	125.3 ± 0.4	0.02	0.91
Diastolic blood pressure (mm/Hg)	64.8 ± 2.1	72.1 ± 0.3	< 0.0001	0.13
Smoking status (%)			< 0.0001	0.06
Current smoker	10.5	20.8		
Former smoker	52.8	30.6		
Never smoker	36.7	48.6		
Alcohol consumption (%)			0.65	0.27
Binge drinker	18.1	20.1		
Heavy drinker	4.7	6.9		
Moderate drinker	46.4	39.2		
Non drinker	24.9	20.6		
Unknown/missing	5.9	13.2		
Physical activity (%)			0.20	0.69
Moderate or vigorous	45.3	55.4		
None	54.7	44.6		
Prevalent diabetes^c^ (%)			0.0002	0.004
No	65.5	87.7		
Borderline	5.5	2.0		
Yes	29.0	10.4		
History of cancer (%)			0.07	0.71
No	80.0	88.2		
Yes	20.0	11.8		
History of cardiovascular disease^d^ (%)			0.20	0.12
No	83.0	88.2		
Yes	17.0	11.8		

All proportions and means (± standard errors) are weighted estimates of the US population characteristics, taking into account the complex sampling design of the National Health and Nutrition Examination Survey^20^.

^a^P values for differences from survey-weighted logistic regression models.

^b^P for difference tested in the entire population; age-stratification only used for descriptive purposes.

^c^Participants were asked by an interviewer whether they had ever been told by a doctor or health professional that they had diabetes; borderline status assigned by interviewers depending on the participants’ response.

^e^Cardiovascular disease includes: Self-reported heart failure, coronary heart disease, angina pectoris, heart attack, and stroke.

Ophthalmologic characteristics of the study population are shown in Table 2. Glaucoma patients had poorer visual acuity, with an average logMAR value of 0.20 ± 0.02 compared to 0.13 ± 0.003 among participants without glaucoma, but this difference was not statistically significant upon adjustment for age. Prevalent late AMD (2.2% vs. 0.6%) and a history of cataract surgery (34.4% vs. 8.6%) were more common among glaucoma patients, whereas the rate of retinopathy was slightly lower (7.4% vs. 9.7%). Again, none of these differences were statistically significant when age was accounted for in logistic regression. Only 55.8% of the study participants, who were diagnosed with glaucoma during the NHANES examinations, had also self-reported a glaucoma, and 22.0% had self-reported having used glaucoma-specific medications.

**Table 2:** Vision and self-reported use of ophthalmic medication of the NHANES 2005–2008 study participants with and without diagnosed glaucoma.

	Participants		*P* for difference^a^	
	With glaucoma (n = 138)	Without glaucoma (n = 5247)	Unadjusted	Age-adjusted
Final FDT^b^ right eye status (%)			< 0.0001	< 0.0001
Insufficient	2.1	0.7		
Normal	51.9	87.7		
Not Done	1.7	0.9		
Positive	27.3	2.5		
Unreliable	17.0	8.3		
Final FDT^b^ left eye status (%)			< 0.0001	< 0.0001
Insufficient	1.1	0.7		
Normal	50.4	81.5		
Not Done	0	0.9		
Positive	30.4	3.5		
Unreliable	18.1	13.4		
Visual acuity (logMAR)				
Right eye	0.22 ± 0.04	0.13 ± 0.003	0.0002	0.43
Left eye	0.17 ± 0.02	0.13 ± 0.004	0.02	0.18
Spherical equivalent^c^				
Right eye	− 0.28 ± 0.25	− 0.44 ± 0.06	0.54	0.45
Left eye	− 0.05 ± 0.24	− 0.43 ± 0.06	0.15	0.13
Average both eyes	− 0.17 ± 0.24	− 0.44 ± 0.06	0.29	0.23
Prevalent retinopathy, any^d^ (%)			< 0.0001	0.38
No	92.6	90.3		
Yes	7.4	9.7		
Self-reported cataract surgery, ever (%)				
No	67.6	91.4	< 0.0001	0.29
Yes	32.4	8.6		
Prevalent late AMD^d^ (%)			0.50	0.16
No	97.8	99.4		
Yes	2.2	0.6		
Self-reported glaucoma (%)			< 0.0001	< 0.0001
No	44.2	95.8		
Yes	55.8	4.2		
Use of any glaucoma medication (%)			< 0.0001	< 0.0001
No	78.0	98.9		
Yes	22.0	1.1		
Use of beta blockers (%)			< 0.0001	< 0.0001
No	94.8	99.8		
Yes	5.2	0.2		
Use of prostaglandin analogs (%)			< 0.0001	< 0.0001
No	90.7	99.5		
Yes	9.3	0.5		
Use of adrenergic agents (%)			< 0.0001	0.0004
No	96.4	99.8		
Yes	3.6	0.2		
Use of carbonic anhydrase inhibitors (%)			0.10	0.82
No	99.7	100		
Yes	0.3	0.0		
Use of combination drugs (%)			< 0.0001	0.0006
No	96.3	99.9		
Yes	3.7	0.1		

All proportions and means (± standard errors) are weighted estimates of the US population characteristics, taking into account the complex sampling design of the National Health and Nutrition Examination Survey.

^a^*P* values for differences from survey-weighted logistic regression models.

^b^FDT: Frequency Doubling Technology.

^c^Calculated as sphere value plus half the cylindrical power value (347 missing values overall, out of which 14 among glaucoma patients).

^d^Prevalent retinopathy and AMD (age-related macular degeneration) derived from retinal imaging^36^.

### All-cause mortality

**Figure 1:**
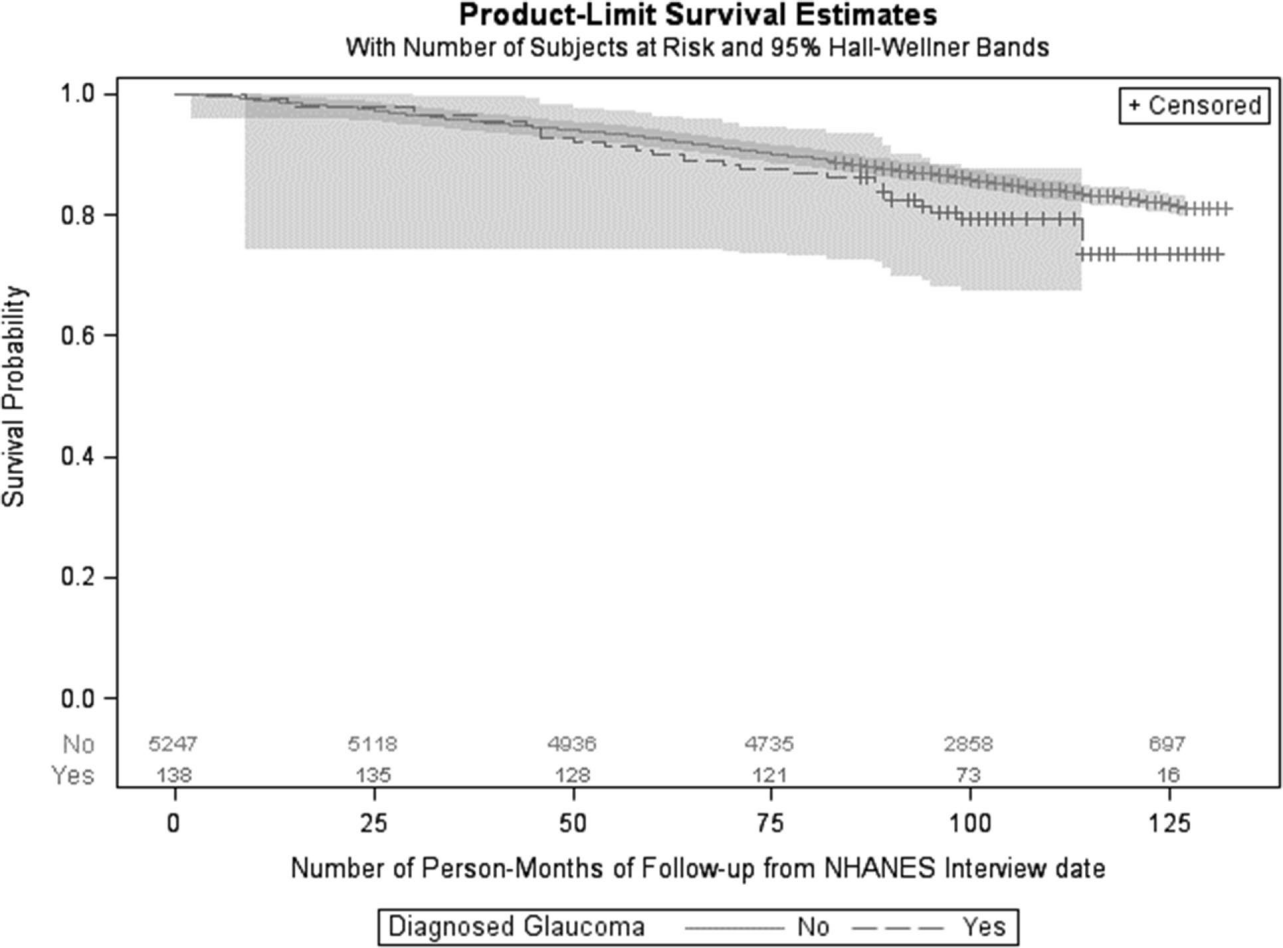
Product limit survival estimates for study participants with and without glaucoma. The grey areas depict 95% Hall–Wellner Bands. Numbers of participants at risk are shown above the x-axis.

After a mean follow-up time of 8.6 years (range: 0.2–11 years), 833 (11.2%) of the study participants died, out of which 30 had a diagnosis of glaucoma at baseline. Results of Cox regression analyses on glaucoma and all-cause mortality are shown in Table 3, and survival curves of participants with and without glaucoma are shown in Fig. 1. The unadjusted regression model revealed a significantly higher risk of death among study participants with glaucoma, with a HR of 2.06 [95% CI: 1.16, 3.66]. However, this association was no longer significant after adjustment for age (HR: 0.74 [0.48, 1.14]). The HR of 0.74 remained the same when further adjusting for sex [CI: 0.46, 1.17]. Adjustment for additional covariates only slightly affected the age-and sex adjusted HR. In a multivariable model with broad adjustment for core socio-economic and lifestyle factors as well as comorbidities and use of glaucoma treatment, the HR for all-cause mortality among participants with diagnosed glaucoma was at 0.83 [0.53, 1.29].

**Table 3:** Associations between diagnosed glaucoma and overall mortality from Cox Proportional Hazards Models.

	Participants		HR (95% CI)			
	Survived (n = 4552)	Died (n = 833)	Model 1^a^	Model 2^b^	Model 3^c^	Model 4^c^
Without diagnosed glaucoma (n = 5247)	89.0% (n = 4444)	11.0% (n = 803)	Ref	Ref	Ref	Ref
With diagnosed glaucoma (n = 138)	78.2% (n = 108)	21.8% (n = 30)	**2.06** (**1.16**, **3.66**)	0.74 (0.48, 1.14)	0.74 (0.46, 1.17)	0.83 (0.53, 1.29)

Participant frequencies are survey-weighted, counts are unweighted. Mortality risk among individuals with glaucoma was statistically signficantly higher at a p-value of 0.02 in Model 1 (HR in bold), but not in any of the other models (all p-values > 0.05).

^a^Unadjusted.

^b^Adjusted for age.

^c^Adjusted for age and sex.

^d^Adjusted for age, sex, ethnicity, marital status, health insurance status, education level, alcohol consumption, smoking status, physical activity, BMI, use of glaucoma treatment, comorbid eyes diseases (age-related macular degeneration, retinopathy, history of cataract surgery), prevalent diabetes, history of cancer, history of CVD.

### Sensitivity analyses

Further adjusting the multivariable Cox regression model 4 shown in Table 3 for prevalent hypertension, or replacing the self-reported variable for prevalent diabetes by measured glycohemoglobin and self-reported diabetes treatment only marginally changed the results, with HRs of 0.85 [0.55, 1.31] and 0.82 [0.54, 1.23]. When restricting the Cox regression analyses to participants older than 65 years at baseline, the association with mortality in the fully adjusted model was highly similar as in the main analysis (HR: 0.80 [0.53, 1.22], also see Additional File 1: Figure S2 for survival curves).

Findings from analyses on cause-specific mortality from competing risk models are shown in Additional File 1: Table S1. Fully adjusted Cox regression analyses did not show significant associations between diagnosed glaucoma and death from cardiovascular disease (HR: 0.72 [0.27, 1.97]), cancer (HR: 0.58 [0.19, 1.75]), or other causes (HR: 0.81 [0.47, 1.39]). There was no indication for heterogeneity of associations between glaucoma status and mortality by survey phase or across strata of ethnicity and sex.

## Discussion

Glaucoma was not associated with all-cause mortality in a representative cohort of the US population older than 40 years. Those with glaucoma were older and therefore had a higher mortality rate but after adjustment for age this association was no longer significant. Overall, our results, based on a representative sample of the US population with high quality data on eye health, strongly indicate that glaucoma is not an independent risk factor for mortality.

The hypothesis that glaucoma may be part of a “systemic process” associated with increased mortality is long-standing, initially motivated by findings from cohorts based on life insurance records relying on self-reported glaucoma diagnosis^8^. Such populations are non-representative and self-report may be indicative of greater use of healthcare which can be related to having other illnesses. This could also explain the strong association reported in a recent study using health insurance claims in Taiwan^11^. The finding could at least partly be explained by the selection of a control group with no ocular diseases. Such a group likely excludes individuals with more severe diabetes, as well as possibly those with different environmental exposures (e.g., age-related macular degeneration is associated with smoking).

Another US population-based cohort study (the National Health Interview Survey) reported an increased risk of death among those with self-reported glaucoma. Similar to the concerns noted above, inadequate adjustment for a range of socio-demographic factors and important confounders such as diabetes could explain this finding. By contrast, we were able to use representative data from the general population recruited for NHANES between 2005 and 2008 including glaucoma diagnosis derived from objective and standardized measurements and we adjusted for a wide range of potential confounders that were collected in a consistent and systematic fashion. Other than age, none of these potential confounders substantially affected the association between glaucoma and mortality.

Another possible explanation for putatively higher mortality rates among patients with glaucoma is the use of certain glaucoma-specific medications^8^. Yet, studies have been very inconsistent^15–18^, with one cohort even suggesting improved survival among users of glaucoma-specific drugs^15^. The authors of the latter study among members of a managed care program discussed that the inverse association they observed with mortality may be related to systemic blood-pressure lowering properties, particularly of β-antagonists, or related to confounding due to differential access to care^15^. Again, however, the present analysis from the representative population-based NHANES cohort did not suggest that medication use may affect associations between glaucoma and mortality.

Several limitations of the present analysis need to be acknowledged. While the NHANES cycles 2005–2006 and 2007–2008 included standardized eye examinations facilitating an expert-based assessment of glaucoma, stereoscopic images were not obtained^7^. Moreover, “true” perimetry was not carried out, and there was no measurement of intraocular pressure. Objective data on the history of glaucoma surgery were not available nor was the onset of the diseases surveyed. Finally, we included all forms of glaucoma in our definition and it may be that POAG alone may have a different association with mortality. Nevertheless, we believe that the use of standardized eye examinations from a representative population-based sample and the comprehensive information on a wide range of possible confounders were particular strengths of the present study. From our point of view, it is unlikely that more detailed ophthalmologic examinations would have led to different results than those presented here, given that optic nerve images were graded by three independent experts and that the final diagnosis was derived from a standardized algorithm^7^. Moreover, the benefit of stereoscopic over monoscopic images for glaucoma assessment has been reported to be minor^19^.

In summary, the present analyses of representative data from the NHANES cycles 2005–2006 and 2007–08 based on expert glaucoma grading did not provide evidence for an increased mortality risk among glaucoma patients. In fact, in those with glaucoma the higher mortality rates were fully explained by older age. Our findings imply that an unknown, systemic underlying condition to cause increased mortality among glaucoma patients may not exist. While higher diabetes rates among glaucoma patients are apparent, the present data suggest that they do not necessarily lead to higher mortality in this group. Overall, the finding that glaucoma is not associated with lower life expectancy may be a re-assuring fact for doctors to communicate to glaucoma patients.

## Methods

### Sample and population

The NHANES is a health and nutritional survey program in the US (https://www.cdc.gov/nchs/nhanes/index.htm, last accessed 2020-05-20) that provides representative data for the non-institutionalized US population. Since 1999, regular data collection in two-year periods is carried out. In each period, approximately 5,000 persons from 15 counties across the country are examined. A multistage probability sampling design is used to select a representative sample of the noninstitutionalized population of the US^20^. Data is collected via questionnaire-based personal interviews at the participant’s home, followed by a visit of a mobile examination center. The NHANES survey cycles 2005–2006 and 2007–2008 included both ophthalmic examinations and retinal imaging of all participants aged 40 years and older. Our statistical analyses are based on the NHANES public use files of these survey cycles, restricted the analyses to participants aged at least 40 years (https://wwwn.cdc.gov/nchs/nhanes/continuousnhanes/default.aspx, last accessed 2020-05-20). All methods were performed in accordance with the relevant guidelines and regulations, and the protocols for the conduct of the NHANES survey protocols were approved by the National Center for Health Statistics (NCHS) Research Ethics Review Board. Informed consent was obtained from all participants^21^.

### Ophthalmic variables

Every participant aged 40 years and older had non-mydriatic 45-degree fundus photographs of the macula and optic disc of both eyes during the NHANES cycles 2005–2006 and 2007–2008. Participants could be excluded from retinal imaging due to blindness (i.e., unable to see light with both eyes open), eye infections, or eye patches on both eyes. Retinal images were first graded by experts at the University of Wisconsin with respect to diabetic retinopathy, age-related macular degeneration, vertical cup-to-disc-ratio (vCDR) and other retinal conditions and diseases (https://wwwn.cdc.gov/nchs/nhanes/2005-2006/OPXRET_D.htm, last accessed 2021-03-16). For the present analysis, participants with vCDR values < 0.6 for both eyes were considered as not having glaucoma, in line with an important previous publication on the prevalence of glaucoma in NHANES form 2016^14^. This cut-point was applied in the analyses for the 2016 paper based on findings of a population-based study on glaucomatous optic neuropathy^22^, and used here for consistency. In 2012, optic disc images with a vCDR grading ≥ 0.6 were re-evaluated at the Wilmer Eye Institute of the Johns Hopkins University School of Medicine by three glaucoma specialists to determine the presence or absence of glaucoma based on clinical judgement of optic disc features (including image quality, notching of the neuroretinal rim, excavation of the optic cup, vCDR, optic disc hemorrhage, tilting of the disc, and relative disc size). Glaucoma was present if the consensus assessment was probable or definite in at least one eye. To determine what proportion of those with vCDR < 0.6 had glaucoma, 180 individuals were graded and the rate was determined to be low (1.6%). Further details about the glaucoma grading process can be found elsewhere^7,14^.

Participants underwent two visual field tests per eye with the Matrix FDT (Carl Zeiss Meditec, Dublin, CA, USA), using the 19-point suprathreshold screening test “N-30-5”^23^. Exclusion criteria were the same as for retinal imaging described above. Presence of visual field defects was determined if at least two abnormal field results (< 1% threshold level) were detected in the first and two in the second test, and if at least one of the detected defects was located in the same position in both tests (“2–2–1 algorithm”). FDT perimetry was not used for the definition of glaucoma, since it was found to be of poor performance in people with no prior experience with visual field testing^7,24^.

Non-cycloplegic autorefraction was obtained using the average of three consecutive measurements (Nidek ARK-760A, Nidek Co. Ltd., Tokyo, Japan). We calculated spherical equivalent (SE) as sphere value plus half the cylindrical power. Presenting distance visual acuity was tested with participants’ own glasses or contact lenses, if available. We transformed visual acuity measurements from Snellen equivalent to logMAR^25^. The category “20/200 + ” was set to 1.1 logMAR.

With respect to ophthalmic comorbidities, data on diagnosed prevalent retinopathy and age-related macular degeneration (AMD) were used^26,27^, while data on the history of cataract surgery was obtained based on self-reports^28,29^.

### Mortality follow-up

Information on vital status, follow-up time, and cause of death was retrieved from the 2015 public-use Linked Mortality Files (LMF) provided by the National Center for Health, and merged with the NHANES data via probabilistic linkage^30,31^. In brief, this linkage procedure is based on a combination of matching criteria (Social Security Number; first name/initial, last name/initial, and birth surname; year, month, and day of birth; sex; race; state of birth) and has been shown to provide accurate results^30^. For analyses on mortality, follow-up time was calculated as the time between baseline and death, or time between baseline and Dec 31, 2015 for participants, who were not matched to a death certificate and thus considered alive.

### Other variables

Covariate datasets were downloaded for the NHANES survey cycles 2005–2006 and 2007–2008^28,29^. Information on age, sex, ethnicity, income, education, medication use, comorbidities and health-related lifestyle factors was self-reported. Anthropometric parameters and blood pressure values were obtained during examinations by trained personnel. Sex-specific categories of self-reported average daily alcohol consumption (non-drinker, moderate drinker, binge drinker, heavy drinker) were used in a similar manner as previously reported by Agrawal et al.^32^. Self-reported information on physical activity was classified based on categories (moderate/vigorous vs. none) proposed by Vasquez et al.^33^. Self-reported smoking status was classified as follows: Never-smokers (less than 100 cigarettes ever), former smokers (no current smoking, but smoked more than > 100 cigarettes in the past), and current smokers^34^. Health insurance categories were combined (none, private, government, government and private) as in a previous NHANES publication on glaucoma prevalence by Gupta et al.^14^.

### Statistical analysis

Out of 5,705 of the participants of the NHANES survey cycles 2005–2006 and 2007–2008 in the age range between 40 and 85 years, who took part in the retinal imaging examinations, data on vertical cup to disc ratio was missing for 210 participants (no available image for either the left or the right eye), and no information on vital status was available for one participant. A glaucoma diagnosis was not possible for 109 participants due to poor image quality, leaving a final analytical sample for the present analyses on glaucoma and mortality of n = 5385 (see Additional File 1: Figure S1). In this analytical sample, there were sporadic missing values for health insurance membership (n = 5), education level (n = 1), marital status (n = 2), smoking status (n = 3), BMI (n = 41), prevalent diabetes (n = 6), and prevalent cardiovascular disease (n = 37), which were imputed using survey-weighted multiple imputation under a missing at random assumption.

Survey-weighted frequencies (categorical variables) and mean values as well as standard errors (SE, continuous variables) were used to describe characteristics of study participants with and without diagnosed glaucoma. As glaucoma was strongly associated with age, differences in covariates between individuals with and without glaucoma were investigated in both unadjusted and age-adjusted survey-weighted logistic regression models. Survival curves of participants with and without glaucoma were generated by Kaplan–Meier estimates. Survey-weighted Cox proportional hazards regression analyses were carried out to obtain HRs and 95% CIs for mortality among individuals with diagnosed glaucoma compared to those without glaucoma. First, the HR for mortality was estimated from a “crude” model, not adjusted for potential confounders (model 1), followed by analyses adjusting for age and sex (model 2). The full multivariable model (model 3) was additionally adjusted for potential confounders shown in Tables 1 and 2, i.e. key socio-economic factors (ethnicity, marital status, health insurance status, and education level), lifestyle factors (alcohol consumption, smoking status, physical activity, and BMI), use of any glaucoma treatment, comorbid eyes diseases (age-related macular degeneration, retinopathy, history of cataract surgery), and other comorbidities (prevalent diabetes, history of cancer, and history of CVD). These potential confounders were selected by literature review. The rather broad selection of covariates was motivated by the notion that glaucoma could reflect an underlying systemic condition. Given the high number of adjustment factors, we calculated condition indices to assess collinearity in Cox regression analyses, but none of the models revealed condition indices > 5, which would have been suggestive of collinearity^35^. Cause-specific hazards from a competing risk model were estimated in ancillary analyses on mortality due to cardiovascular diseases, cancer, or other causes. SAS 9.4 (Cary, NC, USA) was used for all analyses.

## Supplementary Information

Supplementary Information.
