# Supplementary material for: Glaucoma and mortality risk: findings from a prospective population-based study
Source: Sci Rep. 2021 Jun 3;11:11771. doi: 10.1038/s41598-021-91194-3 (PMC8175711; doi:10.1038/s41598-021-91194-3)
Supplement: Supplementary file 1 — Supplementary Information. [file 41598_2021_91194_MOESM1_ESM.docx]

# Supplementary files


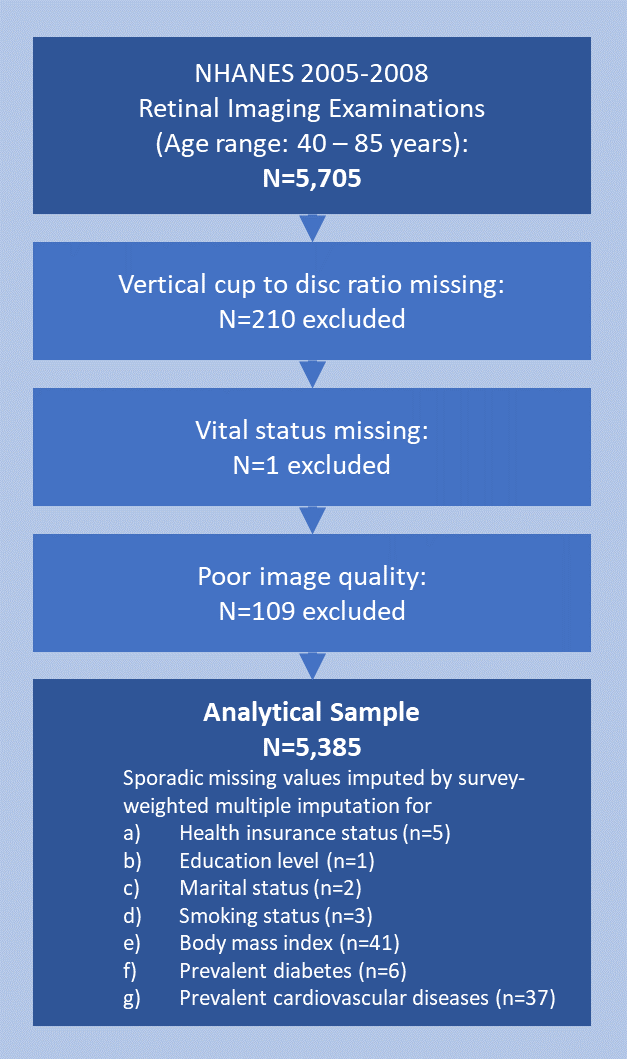


**eFigure 1** Flow chart depicting exclusions and imputed values


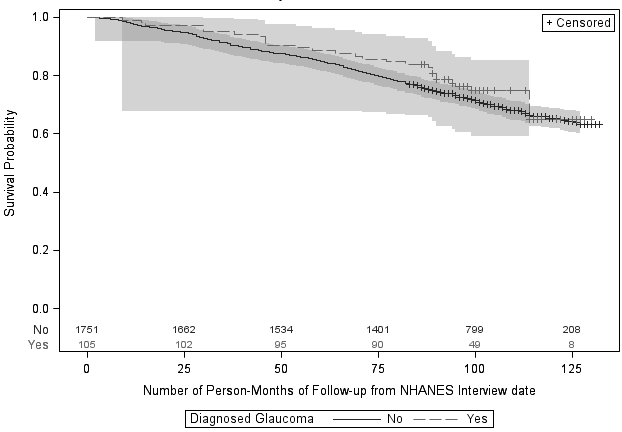


**eFigure 2** Survival curves for study participants with and without diagnosed glaucoma

Product limit survival estimates restricted to participants, who were 65 years and older at baseline. The grey areas depict 95 % Hall-Wellner Bands. Numbers of participants at risk are shown right above the x-axis.

| **eTable 1.** Associations between diagnosed glaucoma and cause-specific mortality from Cox Proportional Hazards Models (cause-specific hazard analyses) | | | | | | |
| --- | --- | --- | --- | --- | --- | --- |
|  | **Participants** | |  | **HR (95% CI)** | | |
|  | Censored | Died |  | Model 1^a^ | Model 2^b^ | Model 3^c^ |
| **Cardiovascular Death** |  |  |  |  |  |  |
| Without diagnosed glaucoma | n=5,088 | n=159 |  | Ref. | Ref. | Ref. |
| With diagnosed glaucoma | n=133 | n=5 |  | 2.16  (0.69, 6.80) | 0. 72  (0.25, 2.02) | 0.72  (0.27, 1.97) |
|  |  |  |  |  |  |  |
| **Cancer Death** |  |  |  |  |  |  |
| Without diagnosed glaucoma | n=5,062 | n=185 |  | Ref. | Ref. | Ref. |
| With diagnosed glaucoma | n=133 | n=5 |  | 1.22  (0.37, 4.00) | 0.57  (0.18, 1.82) | 0.58  (0.19, 1.75) |
|  |  |  |  |  |  |  |
| **Other Causes of Death** |  |  |  |  |  |  |
| Without diagnosed glaucoma | n=4,788 | n=459 |  | Ref. | Ref. | Ref. |
| With diagnosed glaucoma | n=118 | n=20 |  | 2.39  (1.22, 4.68) | 0.79  (0.45, 1.40) | 0.81  (0.47, 1.39) |
| ^a^Unadjusted  ^b^Adjusted for age and sex  ^c^Additionally adjusted for ethnicity, marital status, health insurance status, education level, alcohol consumption, smoking status, physical activity, BMI, use of glaucoma treatment, comorbid eyes diseases (age-related macular degeneration, retinopathy, history of cataract surgery), prevalent diabetes, history of cancer, history of CVD  *Participant counts are unweighted.* | | | | | | |
